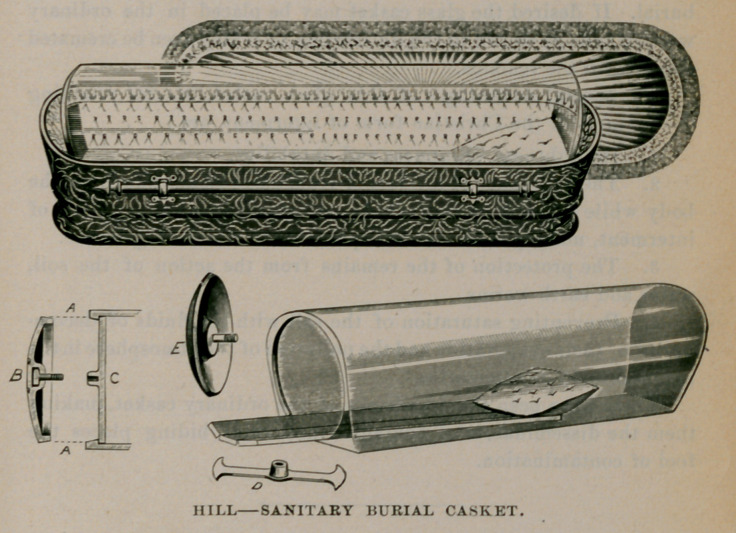# A Sanitary Burial Casket

**Published:** 1895-08

**Authors:** Julian P. Hill

**Affiliations:** Buffalo, N. Y.


### New Inventions.

### A SANITARY BURIAL CASKET.

By JULIAN P. HILL, M. D., Buffalo, N. Y.

THE safest and most humane disposition of the dead has been one of the perplexing problems of sanitarians from time immemorial ; and of all the various methods adopted by civilised tribes or nations, but two have been handed down to us, namely—cremation and interment.

Cremation was a very early and widespread usage of antiquity, dating as far back as the "bronze age," extending down the centuries to the Christian epoch, when it was almost entirely suppressed. Of late years it has been strongly re-advocated from a hygienic and sanitary standpoint, and as vigorously denounced on the ground of sentiment and religion.

HILL—SANITARY BURIAL CASKET.

The prevalent mode of disposing of the dead since the begining of the Christian era has been interment in some field consecrated for this purpose only. The advantages and disadvantages of these two methods are about equal, the safest being cremation, while the most humane and indulgent is burial.

To combine the advantages of the two and minimise their disadvantages has been the aim of the writer in devising the sanitary burial case which is here described for the first time.

This burial casket is constructed entirely of glass, forming a cylinder with a flattened base, closed at one end ; at the other, a circular opening, into which fits a circular end-piece (E). The edges (A) of the end-piece are ground, fitting snugly into the circular opening, and it is held securely by being screwed into a cross-bar (C and D) placed on the inside of the casket. The remains are placed upon a properly draped slab and then introduced into the casket ; the end-piece is screwed into the cross-bar, the ground surfaces come into contact and the body is preserved in an air-tight compartment for burial. If desired the glass casket may be placed in the ordinary wooden case, as shown in the illustration, or it may even be cremated along with the remains.

The special advantages of this glass casket, besides retaining the features of the Christian form of interment, are :

1. Its lightness, cheapness and durability.

2. The avoidance of all danger from gases emanating from the body while exposed to view or during transportation to place of interment, making it almost indispensable in times of epidemic.

3. The protection of the remains from the action of the soil, water and earth-worms.

4. Preventing saturation of the soil with the fluids of decomposition, their evaporation and the pollution of the atmosphere in the neighborhood of cemeteries.

These have been the drawbacks of the ordinary casket, making them the disseminators of contagion and their biding places the foci of contamination.

---

THE RESUSCITATION OF STILL-BORN INFANTS.—Bedford Brown (*Am. Jour. Med. Sciences*) says : During the past three or four years, in several cases of this kind, apparently under the most hopeless circumstances, when all other methods have failed, he has resorted to hypodermatic injections of brandy or whisky with the most satisfactory results. The amount used is five or six drops in first one arm and then in the other, fifteen drops being the largest quantity used in a single case. If the mother has suffered alarming ante-partum hemorrhage, and the infant has been drained of blood before its birth, this method can avail nothing.

## Figures and Tables

**Figure f1:**